# Changes in 12-month outcomes over time for age-related macular degeneration, diabetic macular oedema and retinal vein occlusion

**DOI:** 10.1038/s41433-022-02075-6

**Published:** 2022-05-04

**Authors:** Sanjeeb Bhandari, Vuong Nguyen, Adrian Hunt, Pierre-Henry Gabrielle, Francesco Viola, Hemal Mehta, Les Manning, David Squirrell, Jennifer Arnold, Ian L. McAllister, Daniel Barthelmes, Mark Gillies

**Affiliations:** 1 The University of Sydney, Sydney Medical School, Discipline of Ophthalmology and Eye Health, Save Sight Institute, Sydney, NSW Australia; 2 Department of Ophthalmology, Dijon University Hospital, Dijon, France; 3 Fondazione Cà Granda Ospedale Maggiore Policlinico, University of Milan, Milan, Italy; 4 Department of Clinical Sciences and Community Health, University of Milan, Milan, Italy; 5 Department Ophthalmology Department, Royal Free London NHS Foundation Trust, London, UK; 6 Central Brisbane Eye Clinic, Brisbane, QLD Australia; 7 Auckland District Health Board, Auckland, New Zealand; 8 Marsden Eye Specialists, Sydney, NSW Australia; 9 Lions Eye Institute, The University of Western Australia, Nedlands, WA Western Australia; 10 Department of Ophthalmology, University Hospital Zurich, University of Zurich, Zurich, Switzerland

**Keywords:** Outcomes research, Education

## Abstract

**Objectives:**

To identify whether the outcomes of neovascular age-related macular degeneration (nAMD), diabetic macular oedema (DMO) and retinal vein occlusion (RVO) in routine clinical practice have changed over time.

**Methods:**

We analysed 12-month outcomes in treatment-naïve eyes that started aflibercept or ranibizumab for nAMD (3802 eyes), DMO (975 eyes), Branch RVO (BRVO, 357 eyes), Central RVO (CRVO, 371 eyes) and Hemi-RVO (HRVO, 54 eyes) from 2015 and 2019 tracked in the prospectively designed observational Fight Retinal Blindness! Registry.

**Results:**

The mean VA change at 12-month for each year between 2015 and 2019 remained stable or otherwise showed no discernible trends over time in eyes with nAMD (+3.3 to +6 letters), DMO (+3.6 to +6.7 letters) and RVO (+10.3 to +11.7 letters for BRVO, +5.9 to +17.7 letters for CRVO and 10.2 to 20.7 letters for HRVO). The median number of VEGF-inhibitor injections in eyes that completed 12-month follow-up also remained stable at 8–9 for nAMD, 6–7 for DMO, 7–9 for RVO. Fewer eyes (<one-fourth) that started treatment between 2015 and 2018 and more eyes starting in 2019 did not complete 12-month’s follow-up visit. The mean VA in non-completers at their last visit was higher than that of their baseline visit.

**Conclusions:**

Treatment patterns and outcomes for nAMD, DMO and RVO in routine clinical practice have stabilised over the past 5 years at levels inferior to those reported by the pivotal phase 3 studies. A conscious effort to treat these conditions more intensively, or with longer lasting agents, would likely improve outcomes further in our patients.

## Introduction

Vascular endothelial growth factor (VEGF) inhibitors have been the first-line treatment of neovascular age-related macular degeneration (nAMD), diabetic macular oedema (DMO) and retinal vein occlusion (RVO) since their efficacy was first established in the pivotal clinical trials [1–3]. Studies reported that eyes with nAMD, DMO and RVO received fewer VEGF-inhibitor treatments in routine clinical practice, with inferior outcomes, than those in the clinical trials [4–17]. An earlier analysis from the Fight Retinal Blindness! Registry reported that the 2-year visual outcomes of nAMD in routine clinical practice improved from +2.7 letters for eyes starting treatment in 2007 to +7.8 letters for those starting in 2012 as injection rates over two years increased from 10 in 2007 to 14 in 2012 [18, 19]. The outcomes in eyes that started VEGF inhibitors for DMO in routine clinical practice improved after 2013 because the treatment was started earlier when the visual acuity was better and injections were more frequent than those that started in the previous years [10–12]. Data on more recent outcomes may establish whether treatments have improved further over time and identify areas where they appear suboptimal if they have not. This study aimed to report 12-month outcomes in eyes that started VEGF inhibitors for nAMD, DMO and RVO from 2015 to 2019 in routine clinical practice.

## Methods

### Design, data sources and measurements

Data were collected in the prospectively designed web-based registry for tracking treatment outcomes of macular diseases—The Fight Retinal Blindness (FRB)! Registry [20]. The registry has modules to collect data of eyes that receive treatment for nAMD, DMO and RVO in routine clinical practice.

The data recorded at each clinical visit include the number of letters read on a logarithm of the minimum angle of resolution (logMAR) VA Chart, the activity of the choroidal neovascular membrane (CNV) in eyes with AMD and central subfield thickness (CST [µm]) for DMO and RVO assessed using spectral-domain optical coherence tomography (OCT), procedures and ocular adverse events. Enrolment in the audit required a baseline visit when the first injection was administered that had extra data points regarding demographics, type (AMD, diabetes, RVO) and the presence or absence of key risk factors. All treatment decisions, including choice of treatment and frequency of visits, were based on VA and OCT at the discretion of the practitioner in consultation with the patient, thereby reflecting real-world clinical practice.

Participants in this analysis were patients from practices in Australia, France, Italy, Switzerland and the United Kingdom. Institutional approval was obtained from the Royal Australian and New Zealand College of Ophthalmologists Human Research Ethics Committee, the Southeastern Sydney Local Health District Human Research Ethics Committee, the French Institutional Review Board (Société Française d’Ophtalmologie Institutional Review Board), the Ethics Committee of the University of Milan, the Cantonal Ethics Committee Zurich and the Caldicott Guardian at the Royal Free London NHS Foundation Trust. Informed consent (‘opt-in consent’) was sought from patients in France, Italy and Switzerland. Ethics committees in Australia approved the use of “opt-out” patient consent. Data in the registry are anonymized and compliant with the UK Policy Framework for Health and Social Care Research. This study adhered to the tenets of the Declaration of Helsinki.

### Patient selection

Treatment naïve eyes that started ranibizumab (0.5 mg Lucentis; Genentech, Inc., South San Francisco, CA; Novartis, Basel, Switzerland) or aflibercept (2 mg Regeneron Pharmaceuticals Inc., Tarrytown, NY/Bayer, Leverkusen, Germany) for the treatment of nAMD, DMO and RVO from 1 January 2015 to 31 December 2019 allowing the possibility of having at least 12 months of follow-up. Eyes that received fewer than 2 injections were excluded from the analysis. Eyes that completed at least 12 months of visits were defined as ‘completers’. Eyes that did not complete 12 months of observations were defined as ‘non-completers’.

### Outcomes

The main outcome was the mean change in VA at 12 months for each year from 2015 to 2019. Secondary outcomes were the number of injections and visits over 12 months for each year, the proportion of visits that were graded active each year in eyes with nAMD, mean change in CST at 12 months for each year in eyes with DMO and RVO, the proportion of eyes with VA ≥ 69 letters (Snellen equivalent 20/40) and ≤35 letters (20/200) at baseline and 12 months for each year and the proportion of eyes that gained ≥10 letters and those that lost ≥10 letters at 12 months for each year. Other outcomes of interest were the non-completion rates for each year.

### Statistical analysis

Descriptive data included the mean (standard deviation), median (first and third quartiles) and percentages where appropriate. Eyes were considered to have been observed from the first treatment visit up to their 12 months (365 ± 30 days) visit. Paired *t* tests, Wilcoxon signed-rank test, Chi-square and Fisher tests were used as appropriate to compare the changes at 12 months from the baseline. Line graphs were used to visualise changes in VA and CST and bar plots for the number of injections and visits at 12 months.

We used mixed-effects models to compare VA outcomes at 12 months over time. The models were adjusted for age, baseline CST (for DMO and RVO), baseline VA, (fixed-effects), and practice and intra-patient correlation for bilateral cases (random-effects) with year of treatment initiation as a continuous variable. All analysis will be conducted using R statistical software version 4.1.2 (http://www.R-project.org/) with the *glmmTMB* package (V1.1.2.3) for multivariate analysis [21].

## Results

### Study participants

A total of 3802 eyes (3284 patients) with nAMD, 975 eyes (718 patients) with DMO, 357 eyes (351 patients) with branch retinal vein occlusion (BRVO), 371 eyes (368 patients) with central retinal vein occlusion (CRVO) and 54 eyes (54 patients) with Hemi-retinal vein occlusion (HRVO) started VEGF inhibitors whose treatment outcomes were tracked in the registry in the period specified. Data from both eyes of 518 nAMD, 257 DMO, 6 BRVO and 3 CRVO patients were included in the analysis. Table 1 summarises the baseline characteristics of these eyes. The baseline characteristics of eyes for each year from 2015 to 2019 in the disease group of nAMD, DMO, BRVO, CRVO and HRVO are reported in Supplementary Table 1.

**Table 1:** Demographic characteristics.

	nAMD	DMO	BRVO	HRVO	CRVO
Eyes, *n*	3802	975	357	54	371
Patients, *n*	3284	718	351	54	368
Female, *n* (%)	2012 (61)	259 (36)	185 (53)	23 (43)	167 (45)
Right Eye, *n* (%)	1957 (52)	501 (51)	195 (55)	24 (44)	194 (52)
Age years, mean (SD)	80 (9)	63 (12)	71 (12)	73 (11)	72 (13)
Baseline VA letters, mean (SD)	59.2 (20)	65.4 (16.8)	58 (18)	47.9 (23.9)	42 (26)
VA ≥ 69 letters, %	41	56	33	26	17
VA ≤ 35 Less, %	14	7	12	32	39
CST, µm (SD)	–	415 (126)	472 (156)	576 (212)	624 (229)
Lesion, %	CNV lesion type	DMO type	Ischaemia	Ischaemia	Ischaemia, %
	Type 1—41 Type 2—17 Type 3—5 Other—5 Not done—32	Centre-involving—88 Non centre-involving—9 No CSME—3	Macular—5 Peripheral—13	Macular—4 Peripheral—30	Macular—6 Peripheral—21

*nAMD* Neovascular age-related macular degeneration, *DMO* Diabetic macular oedema, *BRVO* Branch retinal vein occlusion, *HRVO* Hemi-retinal vein occlusion, *CRVO* Central retinal vein occlusion, *n* number, *SD* standard deviation, *DR* Diabetic retinopathy, *VA* visual Acuity, *CST* Central subfield thickness, *CNV* Choroidal neovascularization.

### Visual outcomes at 12 months in completers

**Fig. 1:**
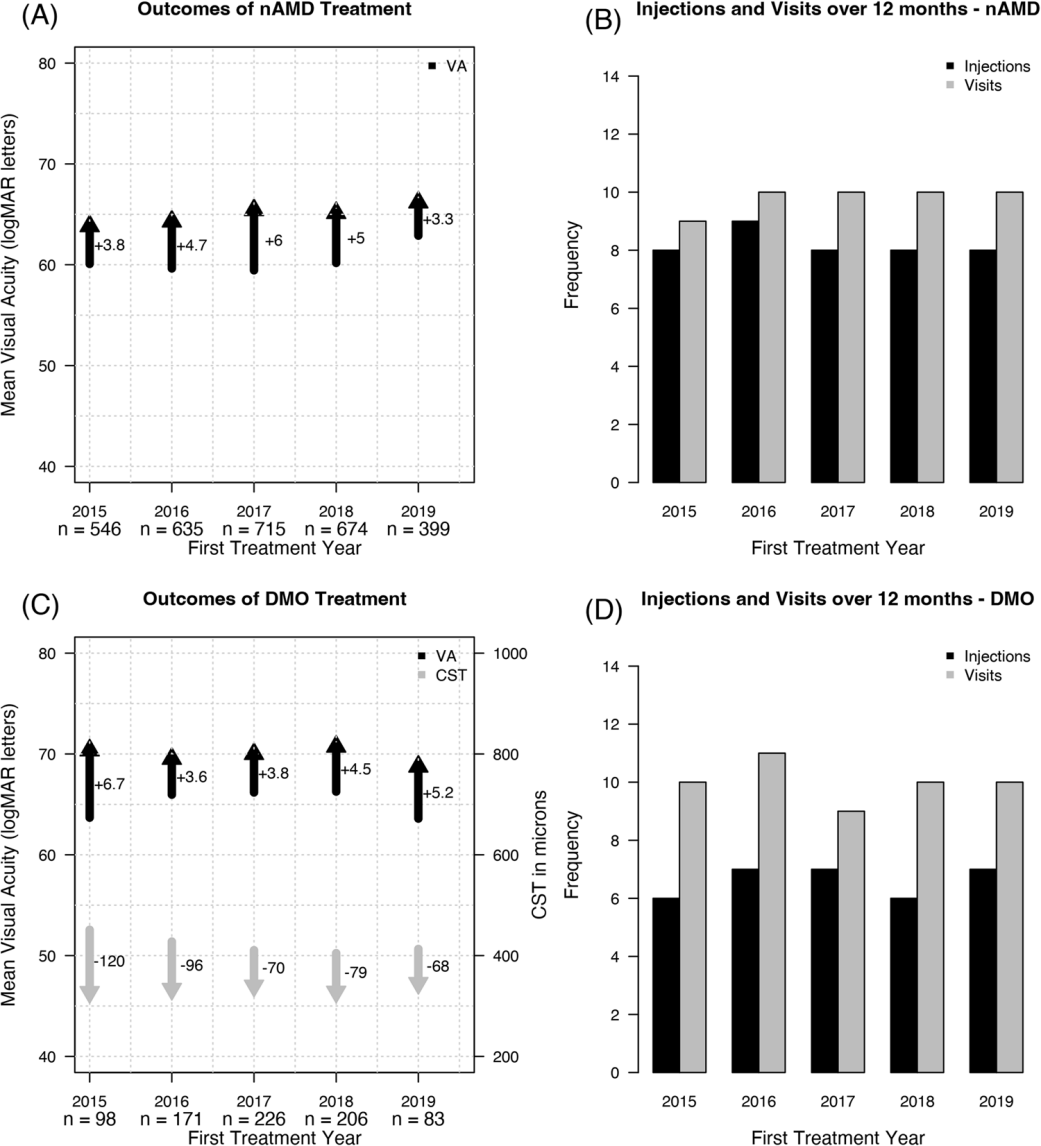
Treatment outcomes in age-related macular degeneration and diabetic macular oedema. **A** Mean change in visual acuity (VA) at 12 months in eyes that completed 12 months of vascular endothelial growth factor (VEGF) inhibitor treatments for neovascular age-related macular degeneration (nAMD). Outcomes are shown by the year of treatment initiation. The base of each arrow represents the mean baseline VA while the point represents the mean final VA. The mean changes in VA are reported next to the arrows. ‘*n*’ represents number of eyes. **B** Bar plot illustrates the median number of VEGF-inhibitor treatments (black bar) and visits (grey bar) over 12 months. **C** Mean change in visual acuity (VA, black) and central subfield thickness (CST, grey) at in eyes that completed 12 months of vascular endothelial growth factor (VEGF) inhibitor treatments for diabetic macular oedema (DMO). Outcomes are shown by the year of treatment initiation. The base of each arrow represents the mean value at baseline while the point represents the mean value at 12 months. The mean changes are reported next to the arrows. ‘*n*’ represents number of eyes. **D** Bar plot illustrates the median number of VEGF-inhibitor treatments (black bar) and visits (grey bar) over 12 months.

Figure 1A illustrates the mean VA at baseline and 12 months, and the mean change in VA over 12 months in eyes with nAMD stratified by the year of starting VEGF-inhibitor treatment. The mean VA at baseline, 59.4–62.9 letters (Snellen equivalent: 20/63–20/60), and the mean gain in VA at 12 months, 3.3–6 letters (*p* = 0.38), for each treatment year tended to be similar (Table 2). The mean VA 12 months after starting treatment was 64–66 letters (Snellen equivalent: 20/50). The proportion of eyes with VA ≥ 69 letters increased (54–60% at 12 months from 41–47% at baseline). Around 27–35% of eyes gained ≥ 10 letters over 12 months (Table 2).

**Table 2:** Outcomes in completers stratified by year of starting treatment.

	2015	2016	2017	2018	2019
**Age-related macular degeneration**					
Completers, *n* (%)	546 (84)	635 (79)	715 (87)	674 (75)	399 (63)
Patients, *n*	512	591	662	612	369
Baseline VA letters, mean (SD)	60.1 (18.7)	59.6 (19.4)	59.4 (20.5)	60.2 (20.4)	62.9 (17.5)
Final VA letters, mean (SD)	63.8 (19.2)	64.3 (20.7)	65.4 (19.9)	65.1 (19.7)	66.2 (18.6)
Change VA letters, mean (95% CI)^a^	3.8 (2.5, 5)	4.7 (3.4, 5.9)	6 (4.9, 7.1)	5 (3.8, 6.1)	3.3 (1.8, 4.8)
Gain ≥10 letters %	31	32	35	31	27
Loss ≥10 letters %	13	12	10	11	12
VA ≥ 69 letters %, baseline/final	41/54	41/59	43/59	44/61	47/60
VA ≤ 35 letters %, baseline/final	12/11	12/12	13/9	13/11	10/8
Injections, median (Q1, Q3)	8 (7, 10)	9 (7, 10)	8 (6, 10)	8 (6, 10)	8 (7, 10)
Visits, median (Q1, Q3)	9 (8, 12)	10 (8, 12)	10 (8, 12)	10 (8, 12)	10 (8, 12)
Active CNV visits, %	36	31	31	24	18
**Diabetic macular oedema**					
Completers, *n* (%)	98 (94)	171 (89)	226 (88)	206 (75)	83 (56)
Patients, *n*	78	133	185	157	63
Baseline VA letters, mean (SD)	63.7 (17.5)	66 (15.8)	66.2 (17.3)	66.3 (16.8)	63.6 (16.7)
Final VA letters, mean (SD)	70.4 (14)	69.5 (16.2)	70 (14.6)	70.7 (17.6)	68.8 (15.8)
Change VA letters, mean (95% CI)^b^	6.7 (3.3, 10.1)	3.6 (1.6, 5.5)	3.8 (2, 5.7)	4.5 (2.6, 6.3)	5.2 (2.1, 8.2)
Gain ≥10 letters %	33	30	24	27	37
Loss ≥10 letters %	7	12	12	7	8
VA ≥ 69 letters %, baseline/final	42/60	53/67	54/66	58/71	53/71
VA ≤ 35 letters %, baseline/final	7/2	5/4	6/3	7/6	7/4
Baseline CST μm, mean (SD)	451 (149)	428 (129)	411 (125)	405 (125)	413 (115)
Final CST μm, mean (SD)	327 (91)	334 (102)	340 (103)	328 (97)	345 (106)
Change CST μm, mean (95% CI)	−120 (−152, −88)	−96 (−116, −77)	−70 (−86, −53)	−79 (−95, −62)	−68 (−92, −44)
Injections, median (Q1, Q3)	6 (4, 8)	7 (5, 9)	7 (5, 8)	6 (4, 9)	7 (5, 9.5)
Additional laser, *n*	7	3	6	2	3
Additional Triamcinolone, *n*	1	1	2	0	0
Additional Ozurdex^®^, *n*	6	11	5	9	8
Visits, median (Q1, Q3)	10 (8, 12)	11 (8, 14)	9 (7, 12)	10 (7.2, 13)	10 (8, 14)
**Branch retinal vein occlusion**					
Completers, *n* (%)	37 (90)	76 (93)	73 (88)	73 (79)	38 (66)
Patients, *n*	37	76	72	73	36
Baseline VA letters, mean (SD)	58.8 (17.9)	56.7 (18.4)	59.9 (16.8)	57.3 (19.2)	63.5 (17)
Final VA letters, mean (SD)	70.5 (14)	67.5 (16.9)	71 (17.6)	69 (17.2)	73.8 (16.3)
Change VA letters, mean (95% CI)^c^	11.7 (5.7, 17.7)	10.8 (7, 14.6)	11.1 (7.4, 14.8)	11.7 (7.5, 15.9)	10.3 (6.2, 14.4)
Gain ≥10 letters %	51	51	59	52	53
Loss ≥10 letters %	8	8	7	9	5
VA ≥ 69 letters %, baseline/final	30/62	29/58	29/75	36/67	47/79
VA ≤ 35 letters %, baseline/final	11/5	12/4	7/4	19/7	5/3
Baseline CST μm, mean (SD)	450 (155)	483 (148)	469 (152)	469 (166)	452 (149)
Final CST μm, mean (SD)	343 (115)	331 (111)	315 (104)	313 (111)	323 (101)
Change CST μm, mean (95% CI)	−108 (−173, −43)	−155 (−195, −115)	−155 (−198, −113)	−156 (−200, −112)	−129 (−182, −75)
Injections, median (Q1, Q3)	7 (5, 9)	8 (5, 9)	7 (5, 9)	7 (6, 9)	8 (6, 8)
Additional macular laser, *n*	2	3	3	1	0
Additional PRP laser, *n*	8	18	6	16	4
Additional Ozurdex^®^, *n*	3	2	2	1	2
Visits, median (Q1, Q3)	9 (8, 11)	10 (8, 11)	10 (8, 11)	10 (8, 12)	9 (8, 11)
**Central retinal vein occlusion**					
Completers, *n* (%)	42 (96)	76 (85)	67 (83)	78 (79)	28 (51)
Patients, *n*	42	75	66	78	28
Baseline VA letters, mean (SD)	40.7 (24)	36 (27.7)	49 (22.4)	45.3 (26.6)	39.7 (28.1)
Final VA letters, mean (SD)	58.5 (25.4)	46.5 (29.8)	63 (22.2)	55.4 (27.2)	45.5 (31.9)
Change VA letters, mean (95% CI)^d^	17.7 (9.2, 26.3)	10.6 (4.7, 16.4)	13.9 (8.3, 19.5)	10.1 (3.4, 16.8)	5.9 (−5.1, 16.8)
Gain ≥10 letters %	71	50	69	44	36
Loss ≥10 letters %	10	15	15	13	25
VA ≥ 69 letters %, baseline/final	14/41	12/30	15/54	24/40	21/32
VA ≤ 35 letters %, baseline/final	45/17	46/37	24/13	35/24	42.9/36
Baseline CST μm, mean (SD)	609 (209)	618 (265)	631 (205)	622 (251)	634 (227)
Final CST μm, mean (SD)	352 (201)	343 (200)	359 (168)	342 (202)	367 (221)
Change CST μm, mean (95% CI)	−260 (−350, −171)	−274 (−348, −201)	−277 (−342, −211)	−290 (−359, −222)	−241 (−368, −114)
Injections, median (Q1, Q3)	8 (6, 10)	7 (5, 9)	8 (6, 10)	8 (5, 10)	9 (7, 9)
Additional macular laser, *n*	0	0	1	0	0
Additional PRP laser, *n*	7	24	14	22	11
Additional Ozurdex^®^, *n*	1	2	3	6	1
Visits, median (Q1, Q3)	10 (9, 13)	11 (9, 13.2)	10 (8, 13)	11 (8, 13)	11 (9, 13)
**Hemi-retinal vein occlusion**					
Completers, *n* (%)	6 (100)	7 (78)	15 (75)	12 (86)	4 (80)
Patients, *n*	6	7	15	12	4
Baseline VA letters, mean (SD)	54.5 (14.4)	47.1 (29.1)	53.6 (21.4)	47.9 (27.1)	48.8 (12.5)
Final VA letters, mean (SD)	73.2 (10.2)	61.4 (29.4)	68.7 (12.1)	68.6 (15.8)	59 (27.4)
Change VA letters, mean (95% CI)^e^	18.7 (−0.8, 38)	14.3 (−5.2, 33.8)	15.1 (4.7, 25.5)	20.7 (10.1, 31.3)	10.2 (−15.3, 35.8)
Gain ≥10 letters %	83	71	60	61	50
Loss ≥10 letters %	17	14	7	0	25
VA ≥ 69 letters %, baseline/final	17/83	43/43	27/60	33/58	0/50
VA ≤ 35 letters %, baseline/final	17/0	43/14	27/0	25/0	25/25
Baseline CST μm, mean (SD)	700 (71)	616 (263)	477 (187)	532 (167)	663 (203)
Final CST μm, mean (SD)	395 (258)	318 (88)	298 (121)	329 (127)	355 (144)
Change CST μm, mean (95% CI)	−305 (−587, −22)	−298 (−535, −61)	−179 (−291, −67)	−203 (−327, −79)	−308 (−706, 90)
Injections, median (Q1, Q3)	10 (8, 10)	8 (7, 10)	7 (5, 9)	7 (6, 9)	9 (8, 9)
Additional macular laser, *n*	0	0	1	0	0
Additional PRP laser, *n*	1	6	7	3	0
Additional Ozurdex^®^, *n*	0	1	0	0	0
Visits, median (Q1, Q3)	10 (9, 11)	12 (10, 13)	9 (8, 13)	9 (9, 11)	9 (8, 10)

We used mixed-effects models to compare VA outcomes at 12 months over time. The models were adjusted for age, baseline CST (for DMO and RVO), baseline VA, (fixed-effects), and practice and intra-patient correlation for bilateral cases (random-effects) with year of treatment initiation as a continuous variable.

*n* Number, *VA* Visual Acuity, *SD* Standard Deviation, *CI* Confidence Interval, *CST* Central Subfield Thickness, *Q1* First Quantile, *Q3* Third Quantile, *PRP* Panretinal photocoagulation.

^a^*p* = 0.38.

^b^*p* = 0.48.

^c^*p* = 0.96.

^d^*p* = 0.98.

^e^*p* =  0.96.

Eyes with DMO tended to have a similar mean change in VA (+3.6 to +6.7 letters, *p* = 0.48) at 12 months from a similar mean VA at baseline of 63.6–66.3 letters (Snellen equivalent: 20/50) when they started treatment (Fig. 1C, Table 2). The mean VA at 12 months from the start of treatment for each year was 69–71 letters (Snellen equivalent: 20/40). More than three-fourths (60–71%) eyes achieved VA ≥ 69 letters over 12 months (Table 2). Almost a quarter to one-third (24–37%) eyes gained ≥10 letters at 12 months (Table 2).

**Fig. 2:**
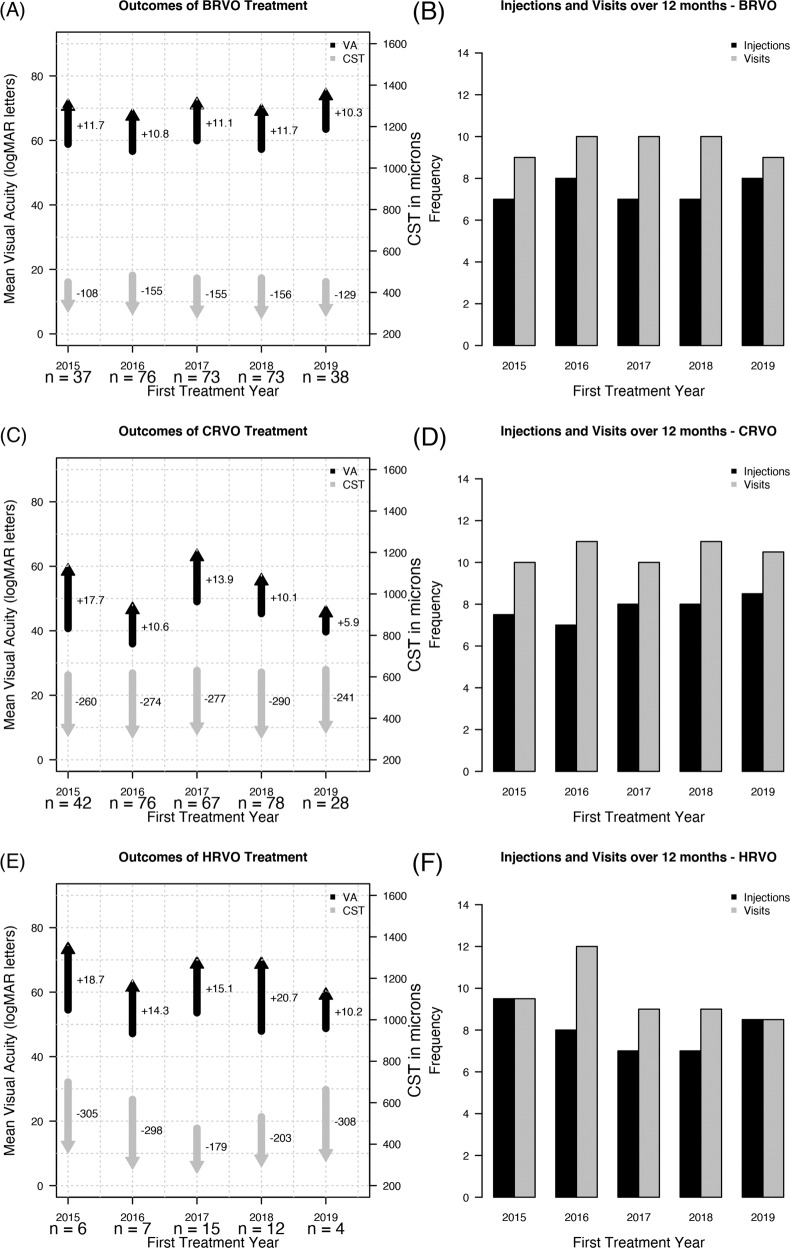
Treatment outcomes in retinal vein occlusion. Mean change in visual acuity (VA, black) and central subfield thickness (CST, grey) in eyes that completed 12 months of vascular endothelial growth factor (VEGF) inhibitor treatments for branch retinal vein occlusion (BRVO, **A**), central retinal vein occlusion (CRVO, **C**) and hemi-retinal vein occlusion (HRVO, **E**). Outcomes are shown by the year of treatment initiation. The base of each arrow represents the mean value at baseline while the point represents the mean value at 12 months. The mean changes are reported next to the arrows. Bar plot illustrates the median number of VEGF-inhibitor treatments (black bar) and visits (grey bar) over 12 months for the BRVO (**B**), CRVO (**D**) and HRVO (**F**) cohorts.

The mean change in VA at 12 months in eyes with BRVO that started VEGF inhibitors in 2015 to 2019 is illustrated in Fig. 2A. The mean change in VA at 12 months was +10.3 to +11.7 letters from a similar mean VA at baseline of 56.7–63.5 letters (Snellen equivalent: 20/70–20/60, *p* = 0.96, Table 2). The mean VA at 12 months from the start of VEGF-inhibitor treatment was 68–74 letters (Snellen equivalent: 20/40, Table 2). The proportion of eyes with VA ≥ 69 letters at 12 months increased (58–79%). More than 50% of eyes gained 10 or more letters (Table 2).

Figure 2B shows the mean change in VA at 12 months in eyes that started VEGF inhibitors for CRVO. The mean VA change in CRVO eyes ranged from +5.9 to +17.7 letters from the mean VA of 36–45.3 letters (Snellen equivalent: 20/200–20/125) at baseline (Table 2). The proportion of eyes with VA ≥ 69 letters at 12 months increased from the baseline while those with VA ≤ 35 letters decreased (Table 2). The mean VA at 12 months improved to 45.5–63 letters (Snellen equivalent: 20/160–20/63; *p* = 0.98, Table 2).

Eyes with HRVO at baseline had a similar trend to those of CRVO eyes (Table 1, Supplementary Table 1). The mean VA change at 12 months in eyes with HRVO was +10.2 to +20.7 letters from a mean VA of 47.1–54.5 letters (Snellen equivalent: 20/125–20/80), *p* = 0.96, at baseline (Table 2). The proportion of eyes with VA ≥ 69 letters at 12 months ranged from 43–80% while those with VA ≤ 35 letters was less than a quarter (Table 2).

### Macular thickness

There were no consistent trends in the mean CST reductions (−120 to −68 µm) at 12 months from the baseline (mean CST: 405–451 µm) or the mean CST at 12 months (327–345 µm) from 2015–2019 in eyes with DMO (Table 2, Fig. 1C).

Eyes with RVO tended to have a stable CST outcome between 2015 and 2019 with a mean CST change (−156 to −108 µm for BRVO, −290 to −241 µm for CRVO and −308 to −179 µm for HRVO) at 12 months from the start of VEGF-inhibitor treatment for each year from the baseline CST (mean CST: 450–483 µm for BRVO, 342–367 µm for CRVO and 298–395 for HRVO; Fig. 2 and Table 2).

### Treatments and visits

The median number of VEGF-inhibitor injections for nAMD in eyes completing 12-month observation from the start of treatment for each year appeared to be stable with a median of 8–9 injections from a median of 9–10 visits (Fig. 1B, Table 2). Similarly, eyes with DMO received a median of 6–7 VEGF-inhibitor injections over the 12 months from a median of 9–11 visits (Fig. 1D) with no clear trends indicating an increase in the number of injections over time. A few eyes in each treatment year required additional treatments, macular laser sessions and intravitreal steroid injections (triamcinolone and Ozurdex^®^ [Allergan Inc., Irvine, CA], Table 2). The number of VEGF-inhibitor treatments in eyes with RVO tended to be similar each year with a median injection of 7–8 injections from a median of 9–10 visits for BRVO (Fig. 2B), 7–9 injections from 10 to 11 visits for CRVO (Fig. 2C) and 7–10 injections from 9 to 12 visits for HRVO (Fig. 2F). A few eyes with RVO required additional treatments, laser (macular, sectoral) and steroid injections (triamcinolone, Ozurdex^®^), over 12 months (Table 2).

### Activity of lesions

The proportion of visits when eyes with nAMD that completed 12-month visit was graded as active tended to decrease over time from 36% of eyes starting treatment in 2015 to 18% in 2019 (Table 2).

### Non-completion rate at 12 months

**Fig. 3:**
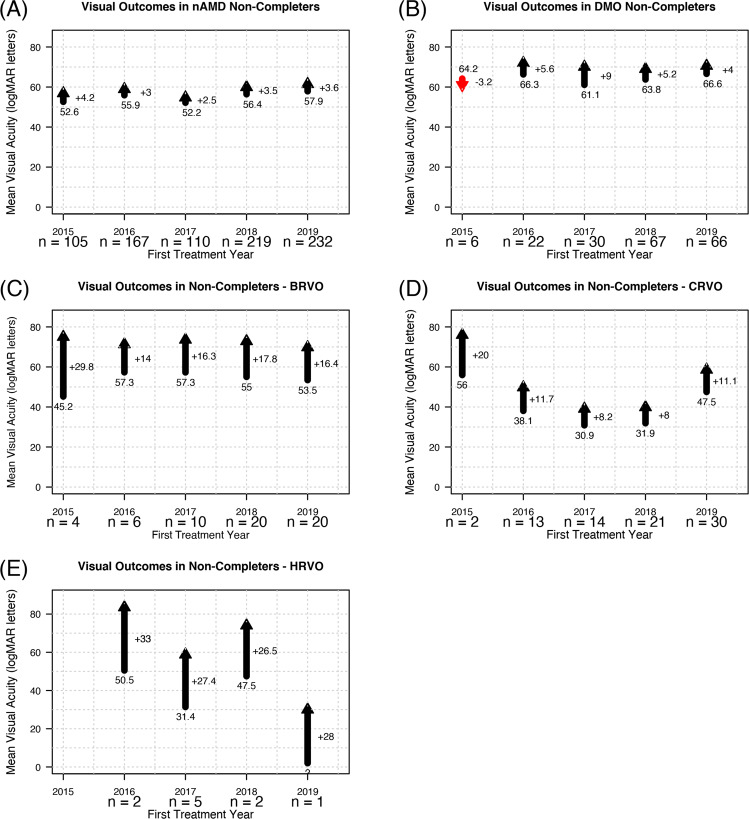
Outcomes in non-completers. Mean change in visual acuity in non-completers from baseline (base) to their last observed visit (point) in eyes that started vascular endothelial growth factor (VEGF) inhibitor treatments for (**A**) neovascular age-related macular degeneration (nAMD), (**B**) diabetic macular oedema (DMO), (**C**) Branch retinal vein occlusion (BRVO), (**D**) Central retinal vein occlusion (CRVO) and (**E**) Hemi-retinal vein occlusion (HRVO). Outcomes are shown by the year of treatment initiation.

Almost a quarter of eyes that started VEGF inhibitor for nAMD during 2015–2018 and a slightly larger proportion (37%) in 2019 did not complete 12 months of observations (Fig. 3A). The mean VA in these eyes at their last visit was better than at the start of treatment and the mean change in VA at dropout for each treatment start year tended to be similar, +2.5 to + 4.2 letters (Fig. 3A).

Six eyes (6%) that started VEGF inhibitor for DMO in 2015 were lost to follow-up before completing 12 months of observations after losing a mean of 3.2 letters from a mean baseline VA of 64.2 letters (Fig. 3B). Fewer than quarter eyes that started DMO treatment in 2016–2018 were lost before the 12-month visit while the non-completers in 2019 increased to 44% (Table 2). These eyes at their last visit had a mean VA change of +4 to +9 letters from 61.1 to 66.3 letters mean VA at baseline (Fig. 3B). Their mean VA at their last visit was >69 letters (20/40) (Fig. 3B).

Fewer than 20% of eyes that started VEGF inhibitors for BRVO and CRVO between 2015 and 2018 were lost over the first 12 months of treatment. Almost half (44% for BRVO and 49% for CRVO) of those that started treatment in 2019 were lost before completing 12 month’s visits. Less than a quarter of eyes with HRVO were lost before 12-month visit (Table 2). The mean change in VA at the last visit from baseline in BRVO non-completers was +14 to +30 letters and their mean VA at the last visit was >69 letters (Fig. 3C). Non-completers in the CRVO group had a mean VA change of +8 to +20 letters at the last visit from the baseline (Fig. 3D) while those in the HRVO gained a mean of 26.5–33 letters (Fig. 3E). The mean VA at the last visit in the CRVO and HRVO non-completers was better than at the start of their treatment.

## Discussion

This analysis of data from routine clinical practice that were collected by a prospectively designed registry for tracking treatment outcomes of macular diseases found that the yearly outcomes of nAMD, DMO and RVO that started VEGF inhibitors between 2015 and 2019 were reasonably good. The visual acuity at baseline for all retinal conditions remained static and the mean visual and anatomical outcomes at 12 months only varied slightly across the years but otherwise did not show any noticeable trends, although the level of CNV activity in eyes with nAMD did decrease over time. The number of injections over 12 months did not increase but remained steady at 8–9 injections for nAMD, 6–7 for DMO, and 7–8 for RVO. These data indicate that the 12-month outcomes of nAMD, DMO and RVO in routine clinical practice have stabilised over the past 5 years despite still being inferior to the outcomes reported by the pivotal clinical trials.

Previous observational studies that evaluated the outcomes of VEGF inhibitors for nAMD found that the visual outcomes in routine clinical practice were inferior to those of the clinical trials with fewer treatments [4, 5, 22, 23]. A study from the FRB! Registry found that the treatment frequency for nAMD in routine clinical practice increased from 2007 to 2012 which resulted in an improvement in the mean change in VA over time [19]. Eyes that started VEGF inhibitors for nAMD in another study of routine clinical practice during 2014 gained a mean of 3.7–4.2 letters at 12 months from a mean of 59 letters at baseline after a mean of 8 VEGF-inhibitor treatments [24]. Studies from the FRB! DMO Registry found that eyes that started treatment from 2009 to 2012 achieved lower 12-month mean VA gains (+2.3 letters from 66 letters at baseline) from fewer treatments (median of 4 injections over 12 months) than those that started treatment after 2013 (+3.1 to +5.4 letters from 64.7–67.8 letters at baseline, median of 6 injections) [10–12]. The present study provides evidence that the treatment outcomes have not impoved further since then and are still inferior to those of clinical trials in which selected patients are managed under a strict protocol regimen.

Eyes presenting with better VA tend to have lower VA gains but are more likely to achieve better vision with treatment [10, 25]. The mean VA at baseline in eyes with nAMD (59 letters), DMO (65 letters) and BRVO (58 letters) in the present study was higher than in those eyes that received VEGF inhibitors in their registrational trials [22, 23, 26–30]. The mean VA at 12 months and the proportion of eyes with VA ≥ 20/40 at 12 months in the present study were similar to those of the registrational trials suggesting that the lower gains in the present study may be the result, in part, of the better starting VA. The mean VA at baseline in eyes with CRVO in the present study was lower, macula thinner and the patients were older than those in clinical trials of VEGF inhibitors for the treatment of CRVO [31–33]. These difference in the baseline characteristics likely contributed to the inferior gains in the present study than those in clinical trials.

The observation that outcomes have remained stable in the last five years might indicate a ceiling may have been reached in what can be achieved in routine clinical practice, which remains inferior to the outcomes of clinical trials. Observational studies may produce results that are inferior to randomised clinical trials due to the biases that are an intrinsic part of the latter [34]. The strict inclusion criteria to be eligible for clinical trials may articially inflate outcomes by excluding patients that are more likely to have a poor response such as those with comorbidities or more severe disease. Patient compliance and adherence to strict regimens are also more difficult to achieve in routine clinical practice resulting in fewer injections and subsequently worse outcomes.

The limitations of this study are inherent to those of observational studies. Treatment decisions in routine clinical practice, in contrast to those in the clinical trials, are not adjudicated by a reference centre or guided by study protocols. Selection of cases, treatment regimen and follow-up schedule may also differ from clinical trials and among physicians. Treatment regimen for each of the retinal diseases used by the centres/physicians in this study was not recorded in the registry. We found 21% of eyes that started VEGF inhibitors between 2015–2019 for all retinal diseases were lost to follow-up before the 12 month’s visit, with increased attrition in eyes starting treatment in 2019 which were definitely affected by COVID-19 pandemic. The mean visual acuity at their last observed visit, except in a few DMO eyes that started treatment in 2015, was better than their baseline which suggests that these eyes could have been lost to follow-up for reasons other than poor outcomes. Nevertheless, we have reported the treatment outcomes of VEGF inhibitors for nAMD, DMO and RVO as they are used in routine clinical practice. There is evidence that carefully designed observational studies, such as the present study, do not consistently overestimate the effectiveness of therapeutic agent [35].

Observational studies may be affected by poor data quality. A recent study of real-world outcomes of nAMD from the American Academy of Ophthalmology Intelligent Research in Sight Registry reported that 35% of VEGF-inhibitor treated eyes recorded in the database lacked baseline and 12-month VA data [36]. The FRB! Registry data can only be accepted into the database for analysis after they have been ‘finalised’ which starts a built-in validation process that checks whether all mandatory fields have been completed and the values are within the pre-determined ranges, for example, visual acuity has to be between 0 and 100 letters [20]. The data were available for subsequent analysis and reporting only when the visits were finalised [20].

This study found that treatment outcomes of nAMD, DMO and RVO in routine clinical practice with VEGF inhibitors have stabilised in the last 5 years. The outcomes we observed were reasonably good, but treatment frequency, 8–9 injections for nAMD, 6–7 for DMO and 7–8 for BRVO, has stabilised at a rate that is lower to those of their registrational clinical trials,12 for nAMD [22, 23], 8–12 for DMO [27, 28], 9 for BRVO [29, 30] and 9–10 for CRVO [31–33], with correspondingly inferior outcomes. Boosting injection rates for nAMD, DMO and RVO, or likely longer lasting agents, would be expected to improve outcomes in our patients. Furthermore, the baseline vision does not seem to have improved over time. Starting treatment earlier before significant vision is lost and increasing the availability of rapid access clinics so that our patients are seen and treated promptly could likely improve outcomes in routine clinical practice. Further research is warranted to evaluate whether long-term outcomes have also stabilised, noting that individualised treatment regimens tend to diverge after 12 months and long-term patient compliance remains a significant challenge in routine clinical practice.

## Summary

### What was known before

- VEGF inhibitors are the standard of care for the treatment of nAMD, DMO and RVO.
- Studies reported that the outcomes in eyes receiving VEGF inhibitors for age-related macular degeneration, DMO and RVO in routine clinical practice were inferior to their pivotal clinical trials.

### What this study adds

- Treatment outcomes of nAMD, DMO and RVO in routine clinical practice with VEGF inhibitors have stabilised in the last 5 years.
- The outcomes were reasonably good, but treatment frequency has stabilised at a rate that is lower than those of the clinical trials with correspondingly inferior outcomes.

## Supplementary information

Supplemental Table 1: Baseline characteristics stratified by treatment start year

Fight Retinal Blindness! Investigators

## Data Availability

Data analysed in this analysis cannot be made openly available due to ethical concerns. We encourage to contact the Save Sight Registries at the University of Sydney, Australia (ssi.ssr@sydney.edu.au) for further information about the data and conditions for access.
