# Supplementary material for: Changes in 12-month outcomes over time for age-related macular degeneration, diabetic macular oedema and retinal vein occlusion
Source: Eye (Lond). 2022 May 4;37(6):1145–54. doi: 10.1038/s41433-022-02075-6 (PMC9066999; doi:10.1038/s41433-022-02075-6)
Supplement: Supplementary file 1 — Supplemental Table 1: Baseline characteristics stratified by treatment start year [file 41433_2022_2075_MOESM1_ESM.docx]

| Supplementary table 1. Baseline characteristics stratified by treatment start year | | | | | |
| --- | --- | --- | --- | --- | --- |
|  | 2015 | 2016 | 2017 | 2018 | 2019 |
| Age-related macular degeneration | | | | | |
| Eyes, n | 651 | 802 | 825 | 893 | 631 |
| Patients, n | 613 | 747 | 762 | 802 | 576 |
| Female, n (%) | 369 (60) | 455 (61) | 481 (63) | 509 (64) | 349 (61) |
| Right Eye, n (%) | 344 (53) | 407 (51) | 429 (52) | 455 (51) | 322 (51) |
| Age years, mean (SD) | 80 (8) | 80 (9) | 79 (10) | 81 (9) | 81 (8) |
| Baseline VA letters, mean (SD) | 58.9 (19.2) | 58.9 (19.8) | 58.5 (20.8) | 59.3 (20.8) | 61.1 (19.2) |
| VA ≥ 69 letters, % | 38 | 40 | 41 | 42 | 45 |
| VA ≤ 35Less, % | 13 | 14 | 14.5 | 14 | 13 |
| Lesion type |  |  |  |  |  |
| Type 1, % | 55 | 49 | 42 | 34 | 26 |
| Type 2, % | 26 | 23 | 12 | 15 | 12 |
| Type 3, % | 3 | 4 | 6 | 6 | 7 |
| Other | 8 | 5 | 5 | 4 | 4 |
| Not done | 8 | 20 | 35 | 42 | 52 |
|  |  |  |  |  |  |
| Diabetic macular oedema | | | | | |
| Eyes, n | 104 | 193 | 256 | 273 | 149 |
| Patients, n | 82 | 150 | 208 | 205 | 114 |
| Female, n (%) | 32 (39) | 57 (38) | 67 (32) | 75 (37) | 41 (36) |
| Right Eye, n (%) | 58 (56) | 99 (51) | 132 (52) | 137 (50) | 75 (50) |
| Age years, mean (SD) | 65 (13) | 63 (12) | 63 (12) | 63 (13) | 65 (11) |
| Diabetes duration years. mean (SD) | 16 (11) | 14 (9) | 16 (9) | 15 (9) | 14 (11) |
| Diabetes type |  |  |  |  |  |
| Type 1, % | 11 | 9 | 8 | 7 | 7 |
| Type 2, % | 89 | 91 | 92 | 93 | 93 |
| Diabetes type |  |  |  |  |  |
| Mild, % | 8 | 17 | 18 | 21 | 18 |
| Moderate | 56 | 33 | 35 | 35 | 38 |
| Severe NPDR | 32 | 29 | 29 | 29 | 37 |
| PDR Non high risk | 3 | 13 | 11 | 10 | 4 |
| PDR Non high risk | 1 | 8 | 7 | 5 | 3 |
| Baseline VA letters, mean (SD) | 63.7 (18.2) | 66 (15.4) | 65.6 (17.4) | 65.7 (16.8) | 64.9 (16.7) |
| VA ≥ 69 letters, % | 47 | 54 | 57 | 59 | 58 |
| VA ≤ 35Less, % | 8 | 5 | 6 | 8 | 8 |
| Baseline CST μm, mean (SD) | 448 (146) | 424 (126) | 417 (129) | 395 (118) | 411 (112) |
| DME type |  |  |  |  |  |
| Centre involving | 92 | 91 | 90 | 84 | 84 |
| Non-centre involving | 6 | 6 | 7 | 12 | 13 |
| No DME | 2 | 3 | 3 | 4 | 3 |
| Branch retinal vein occlusion | | | | | |
| Eyes, n | 41 | 82 | 83 | 93 | 58 |
| Patients, n | 41 | 82 | 82 | 93 | 56 |
| Female, n (%) | 18 (44) | 36 (44) | 46 (56) | 56 (60) | 30 (54) |
| Right Eye, n (%) | 27 (66) | 44 (54) | 41 (50) | 49 (53) | 34 (59) |
| Diabetic retinopathy, n (%) | 3 (7) | 2 (2) | 6 (7) | 5 (5) | 5 (9) |
| Hypertension, n (%) | 0 (0) | 1 (1) | 4 (5) | 1 (1) | 0 (0) |
| Age years, mean (SD) | 71 (11) | 71 (11) | 71 (12) | 71 (11) | 72 (12) |
| Baseline VA letters, mean (SD) | 57.5 (19.3) | 56.7 (18.4) | 59.6 (16.3) | 56.8 (18.7) | 60 (18.7) |
| VA ≥ 69 letters, % | 29 | 29 | 29 | 34 | 41 |
| VA ≤ 35Less, % | 12 | 12 | 6 | 17 | 9 |
| CST, µm (SD) | 452 (162) | 484 (144) | 476 (154) | 476 (165) | 458 (162) |
| Ischemia |  |  |  |  |  |
| Macular, % | 5 | 9 | 2 | 2 | 5 |
| Peripheral, % | 15 | 21 | 8 | 12 | 10 |
| Central retinal vein occlusion | | | | | |
| Eyes, n | 44 | 89 | 81 | 99 | 58 |
| Patients, n | 44 | 88 | 80 | 99 | 58 |
| Female, n (%) | 17 (39) | 41 (47) | 36 (45) | 46 (47) | 28 (48) |
| Right Eye, n (%) | 24 (55) | 49 (55) | 49 (61) | 47 (48) | 25 (43) |
| Diabetic retinopathy, n (%) | 6 (14) | 5 (6) | 5 (6) | 7 (7) | 3 (5) |
| Hypertension, n (%) | 1 (2) | 4 (5) | 6 (7) | 1 (1) | 0 (0) |
| Age years, mean (SD) | 70 (13) | 73 (12) | 74 (13) | 71 (13) | 70 (14) |
| Baseline VA letters, mean (SD) | 41.4 (23.7) | 36.3 (27.3) | 45.9 (23.6) | 42.5 (26.6) | 43.7 (28.1) |
| VA ≥ 69 letters, % | 14 | 14 | 14 | 21 | 21 |
| VA ≤ 35Less, % | 43 | 45 | 31 | 38 | 36 |
| CST, µm (SD) | 606 (207) | 629 (255) | 632 (205) | 626 (247) | 609 (206) |
| Ischemia |  |  |  |  |  |
| Macular, % | 5 | 9 | 7 | 4 | 3 |
| Peripheral, % | 11 | 30 | 22 | 19 | 16 |
| Hemi-retinal vein occlusion | | | | | |
| Eyes, n | 6 | 9 | 20 | 14 | 5 |
| Patients, n | 6 | 9 | 20 | 14 | 5 |
| Female, n (%) | 4 (66.7) | 4 (44.4) | 5 (25) | 5 (35.7) | 5 (100) |
| Right Eye, n (%) | 2 (33.3) | 6 (66.7) | 11 (55) | 3 (21.4) | 2 (40) |
| Diabetic retinopathy, n (%) | 0 (0) | 0 (0) | 0 (0) | 1 (7.1) | 0 (0) |
| Hypertension, n (%) | 0 (0) | 0 (0) | 2 (10) | 0 (0) | 0 (0) |
| Age years, mean (SD) | 73.3 (9.4) | 65.6 (7.8) | 76 (11.3) | 73.6 (11.7) | 76.4 (13.5) |
| Baseline VA letters, mean (SD) | 54.5 (14.4) | 47.9 (28.3) | 48 (24.8) | 47.9 (25.1) | 39.4 (23.5) |
| VA ≥ 69 letters, % | 16.7 | 44.4 | 25 | 28.6 | 0 |
| VA ≤ 35Less, % | 16.7 | 44.4 | 35 | 21.4 | 40 |
| CST, µm (SD) | 699.7 (70.6) | 605.3 (247) | 536.1 (245.9) | 542.9 (176.9) | 629.4 (191.4) |
| Ischemia |  |  |  |  |  |
| Macular, % | 0 | 11.1 | 5 | 0 | 0 |
| Peripheral, % | 33.3 | 66.7 | 30 | 14.3 | 0 |
| n – Number, VA – Visual Acuity, SD – Standard Deviation, CI – Confidence Interval, CST – Central Subfield Thickness, DME – Diabetic macular edema, PDR – Proliferative diabetic retinopathy | | | | | |
