# Supplementary material for: Changes in 12-month outcomes over time for age-related macular degeneration, diabetic macular oedema and retinal vein occlusion
Source: Eye (Lond). 2022 May 4;37(6):1145–54. doi: 10.1038/s41433-022-02075-6 (PMC9066999; doi:10.1038/s41433-022-02075-6)
Supplement: Supplementary file 2 — Fight Retinal Blindness! Investigators [file 41433_2022_2075_MOESM2_ESM.pdf]

Fight Retinal Blindness! Investigators:

Auckland District Health Board, New Zealand (Dr D Squirrell); Adelaide Eye & Retina Centre, South Australia (Professor J Gilhotra); Armadale Eye Clinic, Victoria (Dr A Cohn); Auckland Eye, New Zealand (Dr A McGeorge); Australian Eye Specialists (Bacchus Marsh), Victoria (Dr N Jaross); Australian Eye Specialists (Wyndham), Victoria (Dr N Jaross); Blink, Australian Capital Territory (Dr R Barry); Bundaberg Eye Clinic, Queensland (Dr I McLean); Centre Hospitalier de Saint Brieuc, France (Dr T Guillaumie, Dr A Miri); CHU de Dijon, France (Dr P Gabrielle); CHU de Nice Pasteur 2, France (Dr B Walid); Centre Ophtalmologique Vincennes Vision, France (Dr S Tick); Cairns Eye Surgery, Queensland (Dr A Field); Camberwell Retina Specialists, Victoria (Dr S Wickremasinghe); Canberra Hospital, Australian Capital Territory (Dr C Dayajeewa, Dr J Wells, Dr R Essex); Care Foresight, New South Wales (Dr A Dunlop); Casey Eye Centre, Victoria (Dr K Michalova); Caulfield Eye Clinic, Victoria (Dr C Ng); Central Coast Eye Specialist, New South Wales (Dr S Young); Centre Ophtalmologique de l'Ecole Militaire, France (Dr G Mimoun); Centre for Eye Research Australia, Victoria (Professor R Guymer); Clinique Mathilde, France (Dr J Uzzan); Coastwide Eye Surgery, New South Wales (Dr R Ferrier); Crest Eye Associates, New Zealand (Dr J Ah-Chan); Doncaster Eye Center, Victoria (Dr L Chow); Dorset Consultant Center, Victoria (Dr H Steiner); Dr Alex Amini's Practice, Victoria (Dr A Amini); Dr. Phillip Windle, Queensland (Dr P Windle); Eye Associates, New South Wales (Dr M Gillies, Dr A Hunt); Eye Doctors Mona Vale, New South Wales (Dr P Beaumont, Dr L Cottee); Eye Specialists (Kotara), New South Wales (Dr K Lee); Eye Specialists (Nelson Bay), New South Wales (Dr K Lee); Eye Specialists Greensborough, Victoria (Dr L Chow); Eye Surgeons Miranda, New South Wales (Dr A Hunt); Eye Wide Bay, Queensland (Dr Z Louw); Eyemedics (Wayville), South Australia (Dr K Billing, Dr J Chen, Dr S Lake, Dr J Landers, Dr M Perks, Dr R Phillips, Dr D Qatarneh, Dr N Saha); Fondazione IRCCS Ca' Granda Ospedale Maggiore Policlinico, Italy (Dr F Viola); Gladesville Eye Specialists, New South Wales (Dr S Young); Gordon Eye Surgery, New South Wales (Dr S Fraser-Bell); Hawthorn Eye Clinic, Victoria (Dr E Chong, Dr L Chow); Hornsby Eye Specialists, New South Wales (Dr S Lal); Hospital Tor Vergata Roma, Italy (Professor F Ricci); Kiwi Eye, New Zealand (Dr G Wilson); Les Manning, Queensland (Dr L Manning); Lions Eye Institute, Western Australia (Professor I McAllister); Luigi Sacco Hospital - University of Milan, Italy (Dr A Invernizzi); Maison rouge Ophthalmologic center, France (Dr L Castelnovo, Dr G Michel, Dr B Wolff); Marsden Eye Specialists, New South Wales (Dr J Arnold, Dr H Cass, Dr T Tan); Melbourne Retina Associates, Victoria (Dr A Cohn); Midwest Ophthalmology, New South Wales (Dr K Tang); Mona Vale Eye Centre, New South Wales (Dr C Chung); Montpellier CHU, France (Professor V Daien); Mosman Eye Centre, New South Wales (Dr C Chung); Nepean Valley Eye Surgeons, New South Wales (Dr G Banerjee); New England Eye Centre, New South Wales (Dr M Morgan); North Queensland Retina, Queensland (Dr I Reddie); Port Macquarie Eye Centre, New South Wales (Dr J Game, Dr C Thompson); Retina & Macula Specialists (Hurstville), New South Wales (Dr R Chalasani, Dr M Chilov, Dr A Fung, Dr S Nothling); Retina & Macula Specialists (Miranda), New South Wales (Dr R Chalasani, Dr M Chilov, Dr S Nothling); Retina Associates, New South Wales (Dr R Chong, Dr S Fraser-Bell, Dr A Fung, Dr C Younan); Retina Specialists, New Zealand (Dr R Barnes, Dr D Sharp, Dr A

Vincent); Rotorua Eye Clinic, New Zealand (Dr N Murray); Royal Free London NHS Foundation Trust, United Kingdom (Dr H Mehta); San Martino Hospital, Italy (Dr P Monaco); Southern Eye Centre, Victoria (Dr D Louis); Southern Eye Specialists (NZ), New Zealand (Dr S Every); Specialist Eye Group, Victoria (Dr L Chow, Dr A Cohn); St John of God Hospital Geelong, Victoria (Dr P Lockie); Strathfield Retina Clinic, New South Wales (Dr C Chung, Dr J Wong); Sydney Eye Hospital, New South Wales (Dr M Chilov, Dr R Chong, Dr S Fraser-Bell, Dr M Gillies); Tamworth Eye Centre, New South Wales (Dr P Hinchcliffe); University Hospital Zurich, Switzerland (Dr D Barthelmes); University Hospital Maggiore della Carita, Italy (Dr S Vujosevic); Victoria Parade Eye Consultants, Victoria (Professor R Guymer, Dr A Harper, Dr J ODay); Victorian Eye Surgeons, Victoria (Dr A Cohn); Visionary Eye Specialists, New South Wales (Dr C Hooper)
